# Informing quarantine policy for measles control in primary school and daycare settings: insights from a simulation study, Flanders, Belgium

**DOI:** 10.2807/1560-7917.ES.2026.31.22.2500763

**Published:** 2026-06-04

**Authors:** Cécile Kremer, Naïma Hammami, Laura Cornelissen, Heidi Theeten, Toon Braeye, Andrea Torneri, Steven Abrams, Niel Hens

**Affiliations:** 1 Data Science Institute, I-BioStat, Hasselt University, Hasselt, Belgium; 2 Agency for Care and Health, Infection Prevention and Control, Flemish Community, Ghent, Belgium; 3 Department of Epidemiology and Public Health, Sciensano, Brussels, Belgium; 4 Centre for the Evaluation of Vaccination, Vaccine & Infectious Disease Institute, University of Antwerp, Antwerp, Belgium; 5 Global Health Institute, Family Medicine and Population Health, University of Antwerp, Antwerp, Belgium; 6 Centre for Health Economics Research and Modelling of Infectious Diseases, Vaccine & Infectious Disease Institute, University of Antwerp, Antwerp, Belgium

**Keywords:** measles, public health, simulation model, quarantine, post-exposure vaccination

## Abstract

**BACKGROUND:**

With a notification rate of 44.9 per million population in 2024, Belgium was among the countries with the highest measles rate of the European Union/European Economic Area countries. Although the coverage of the first dose of measles–mumps–rubella (MMR) vaccination in Flanders was 96% in 2020, two-dose coverage was below 95%. To minimise the risk of sustained measles transmission in vulnerable settings, such as primary schools with low vaccination coverage or daycares, it is important to quarantine susceptible contacts of a measles case.

**AIM:**

We aimed to identify policies that balance measles outbreak control with societal as well as educational impact of quarantining children.

**METHODS:**

Using a simulation model, we evaluated the impact of different quarantine strategies for measles in primary school and daycare settings in Flanders, Belgium.

**RESULTS:**

We found that shortening the quarantine period from 21 to 18 days only moderately (e.g. 16–37% in primary school) increased the risk of a subsequent infection wave, and the final proportion of later-generation cases remained at most 3.2% of the school or daycare. In settings with a low vaccination coverage (e.g. 20%), multiple independent introductions or a low diagnosis rate (e.g. only half of symptomatic children going to a doctor), the addition of post-exposure vaccination can further reduce transmission risk.

**CONCLUSION:**

We found that quarantine duration could be shorter than the presumed maximal incubation period of 21 days, without substantially increasing the risk of onward transmission. As such, acceptability and compliance with mandated quarantine periods might also increase.

**Key public health message**

**What did you want to address in this study and why?**

Measles is a highly contagious viral disease which can lead to severe consequences. When measles appears in a primary school or daycare, children may have to stay at home for several weeks, which disrupts learning and family life. We investigated whether these long quarantine periods are always necessary. The goal was to stop measles from spreading while keeping the impact on children and parents as small as possible.

**What have we learnt from this study?**

We found that in many cases, children do not need to at stay home for the full 21 days usually recommended, since shorter quarantine periods of 18 days could be enough. Schools with a vaccination coverage above 70% may also need fewer restrictions. In higher-risk situations, for example when the vaccination coverage is only 20%, vaccinating children quickly after exposure can have an additional benefit.

**What are the implications of your findings for public health?**

These findings support a more tailored approach instead of general and often strict quarantine rules for all situations. This makes the rules easier to understand, accept and follow. Informed by our simulation results, measles policy in Flanders was revised to reduce the quarantine period from 21 to 18 days in daycares, and in primary schools with a vaccination coverage below 70%.

## Introduction

Measles is a highly infectious viral disease and was the leading cause of child morbidity and mortality worldwide before the introduction of vaccines [1]. Although Belgium achieved the World Health Organization (WHO) measles elimination status in 2018, the annual number of measles cases has been rising since 2016, with 367 cases notified in 2017 and 480 in 2019 [2]. During the COVID-19 pandemic, the number of notified measles cases in Belgium decreased to 66 cases in 2020 but has been resurging since the easing of pandemic-related restrictions. In 2024, Belgium was one of the European Union/European Economic Area (EU/EEA) countries with the highest measles incidence, with a notification rate of 44.9 per million population [3].

A person with measles in a setting with a relatively large proportion of susceptible individuals, such as primary schools with vaccination coverage below 95% or daycares with infants (i.e. children below the age of 1 year) not yet vaccinated, brings forth a risk of sustained measles transmission and the emergence of potentially large-scale measles outbreaks. In Belgium, the combined measles–mumps–rubella (MMR) vaccine has been part of the routine childhood immunisation programme since 1985 (with the second dose added in 1995) and is offered free of charge. The first MMR dose is recommended at 12 months of age, implying that younger infants are especially vulnerable to infection when exposed [4]. Historically, a second MMR dose has been administered at the age of 8 and 13 years, albeit that recent changes in the schedule have lowered the age of the second MMR dose. Vaccination coverage and measles circulation are monitored through compulsory notification and coordinated surveillance. In Flanders, the most recent vaccination coverage among children was 96.1% for the first dose, and 93.8% for the second dose [3]. However, two-dose coverage, defined as proof of two doses administered to the same child, was only 89%, which is well below the herd immunity threshold of 95% [5]. In addition, although overall MMR coverage has remained high, research on vaccine confidence in Flanders indicates declining perceptions of the importance and safety of vaccines among the public, which contributes to uneven uptake and under-immunised pockets within the population [6]. Several outbreak reports have also linked low local uptake to measles circulation [7,8], consistent with the idea of heterogeneous vaccine acceptance and delays in vaccination as plausible drivers of vulnerability to outbreaks in otherwise highly vaccinated populations [9].

Quarantine of susceptible close contacts of a measles case is an important measure to curb transmission [10]. The optimal duration of quarantine is, however, influenced by viral dynamics. For measles, a maximal incubation period of 21 days has been cited, although this is based on scarce data [11]. In Flanders, before the present study, the policy in primary schools with a first-dose vaccination coverage below 95% and in daycares involved quarantining and vaccinating presumably susceptible children, i.e. those without proof of vaccination or previous infection, who were in the same room as a confirmed measles case during his or her infectious period (i.e. during the 4 days before and after rash onset). Quarantine was applied for 21 days after last exposure and while this reduces transmission risk, it also imposes a substantial societal burden [12]. An optimal quarantine strategy should balance outbreak control with minimisation of disruption to families and education systems. The aim of the present study was to identify policies that balance outbreak control with societal as well as educational impact of quarantining children.

## Methods

### Simulation model

We used a simulation model to investigate the impact of various quarantine policies on onward measles transmission in primary school and daycare settings. In the model, contact between an infected and a susceptible individual leads to transmission according to a time-varying probability that depends on the infected individual’s time since infection. We used a piecewise constant function for the infectiousness profile *i(t)*, with a non-infectious latent period from the time of infection to 5 days before rash onset, followed by an infectious period of 9 days centred around rash onset [13]. We assumed that all infections are symptomatic and that individuals develop first symptoms according to a lognormal incubation period with a median of 12.5 days and standard deviation of 2.65 days, and rash appearing 3–5 days after the first symptoms with uniform probability [11]. We assumed that 90% of the infected individuals will get tested between their first symptoms and 3 days after rash onset, with a higher probability at rash onset. This assumption results in a relatively high diagnosis rate. The sensitivity of PCR tests on saliva was set to 85% if taken more than 4 days before rash onset, and to 95% otherwise [13]. Confirmed measles cases are isolated from detection until 4 days after rash onset, i.e. until the end of their infectious period. If detection occurs at a time when the individual is already quarantined due to exposure to an infected person, the duration of isolation is adjusted based on the time of rash onset. An overview of these disease progression assumptions has been made available in Supplementary Figure S1 and Supplementary Tables S1–S2. We assumed that all adults (i.e. teachers or daycare staff) were immune due to vaccination or natural infection, and the level of immunity among children was varied in different analyses. Considering a vaccine effectiveness of 93% after one dose [14], the proportion of immune children in school years 1–3 (ages 6–9 years) was set to 89%. In school years 4–6 (ages 10–12 years), we assumed that 95% of children were immune due to vaccination or previous infection. Immune children were randomly distributed across year groups. For daycares, we assumed that maternal immunity had already waned for most infants by the time they started attending daycare, which is generally at the age of 3–4 months [15], and we therefore set the immunity level for infants to 10% in the main analysis [16]. The immunity level for children aged 1–3 years was set to 89% in the main analysis. We performed several sensitivity analyses varying the immunity level and disease progression assumptions. The model was implemented in R version 4.4.3 (https://www.r-project.org/).

In the primary school model, we constructed a set of year groups and sampled the size of a year group from a probability mass function informed by a survey on Belgian primary schools (https://www.vlaanderen.be/agodi), until at least 420 children were allocated to these year groups. We assumed that interaction among children could occur within as well as between year groups. The within-group contact rate was set as the group size minus one, meaning that each child would have on average one contact with their classmates each day. The between-group contact rate was set to 2.5 according to the results of a Belgian contact data survey [17], computed as the number of contacts that last less than 1 h [18]. In daycares, not all children attend daily, which was accounted for by randomly selecting 18 children per day from a pool of 36 children in which 30% were assumed to be infants. As in school classrooms, we assumed that contact was possible between all children. For both primary schools and daycares, we included a weekend effect by not allowing any contacts to occur every 6^th^ and 7^th^ day.

### Transmission heterogeneity

Since measles outbreaks in dense, high-contact settings often involve superspreading, where a single infectious individual infects a disproportionately large number of others, we used an individual reproduction number ν to account for variation in infectiousness [19]. Values for ν were drawn from a negative binomial distribution with mean *R_s_* and overdispersion parameter *k*. In the model, the reproduction number was then approximated by the mean number of effective contacts generated by infectious individuals in a fully susceptible population throughout their infectious period, ν = *q* × *c*. Here, *c* is the total number of contacts. Hence, the transmission potential *q =* ν*/c* with *q* ≤ 1. The probability that a contact between a susceptible and infectious individual was effective, i.e. lead to transmission, was then obtained as *q x i(t)* where *i(t)* was the infectiousness profile.

### Post-exposure vaccination

Timely administration of post-exposure MMR vaccination (PEV) can help to interrupt measles transmission and limit outbreak size, and was therefore included in our primary school model. Considering logistical aspects of its implementation, PEV is offered to susceptible classmates of a measles case (i.e. child infected with measles virus) with a delay of 4 days between case detection and vaccination, and we assumed that 90% of susceptible children would accept vaccination. Since this delay causes PEV to always be administered more than 72 h after exposure (i.e. reactive vaccination with the main goal of preventing a new infection wave), infected children experience the current infection as if they were not vaccinated whereas children who were not yet infected were assumed to be protected against future infections after PEV. First exposure was defined as the first day of being in the same room as the detected case during their infectious period. Individuals who received PEV but were later detected as a measles case were treated as a confirmed case, i.e. they were isolated until 4 days after rash onset and their susceptible contacts were quarantined and/or offered PEV. In the main analysis, we assumed that children who had received PEV were not quarantined, regardless of PEV timing. In a sensitivity analysis we investigated the impact of quarantining all individuals even if they had received PEV.

### Scenarios

For logistical reasons, we assumed a delay of 3 days between case detection and quarantining of exposed contacts. We considered the following baseline and alternative scenarios to investigate the impact of different quarantine policies: only isolating confirmed cases (baseline), quarantining susceptible exposed contacts for 21 (A1) or 18 days (A2) after last exposure, and quarantining from day 9 to day 21 (A3) or from day 9 to day 18 (A4) post-exposure. In the primary school setting, while transmission may occur outside of the classroom, only susceptible classmates of a detected case are quarantined. We investigated the impact of the different quarantine policies on several outbreak characteristics including the proportion of non-index cases (i.e. all cases excluding the index case) causing onward transmission, the probability of new infection waves and the proportion of non-index cases infectious after quarantine. Several additional characteristics were investigated and presented in Supplementary Table S3. For each simulation run, a measles outbreak was initiated by infecting one susceptible child in the school or daycare, and transmission was generated until no infected children were left. Each scenario was run 10,000 times. As a first exploration, we investigated the impact of different quarantine durations (ranging from 10 to 21 days) on limiting the outbreak in a primary school for different immunity levels (ranging from 20% to 95%).

## Results

### Optimal quarantine policy in primary schools and daycares

**Figure 1:**
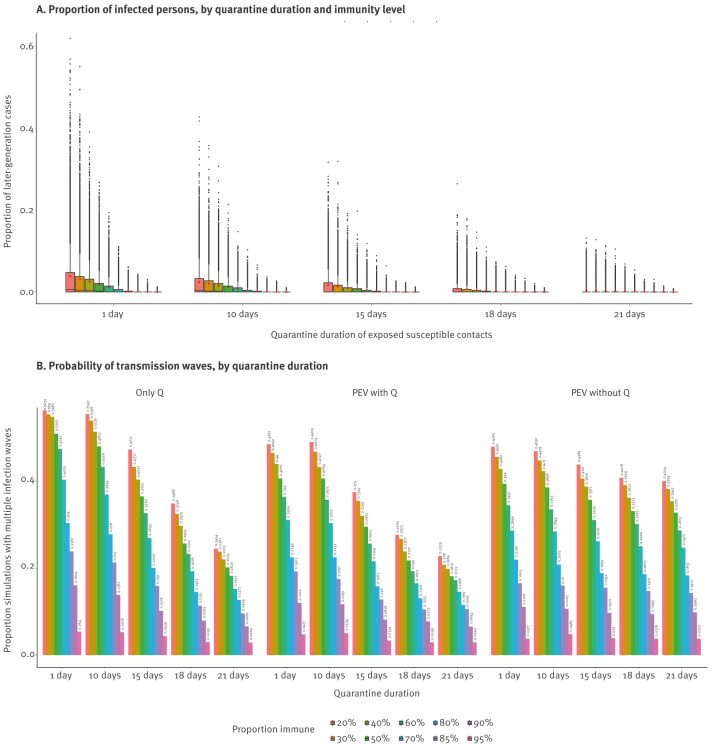
Simulation of transmission of measles virus in primary schools, by quarantine duration and immunity level, Flanders, Belgium

PEV: post-exposure vaccination; Q: quarantine.

As expected, the proportion of the school or daycare that got infected by non-index cases (i.e., later-generation cases), as well as the probability of subsequent infection waves, defined as the proportion of simulations in which later-generation cases occurred, decreased with increasing immunity levels and longer quarantine durations (Figure 1). Based on this first exploration, an immunity level of 70% or higher in primary school was considered to convey an acceptable risk in terms of onward transmission, with an acceptable proportion of later-generation cases of at most 7% without intervention, and was chosen to investigate the impact of quarantine policies in a primary school context in more detail, compared with the main analysis in which 93% of children are immune.

Panel A. Later-generation cases are defined as those from the third generation onward. The red triangles indicate the mean.

Panel B. Proportion of simulation runs for primary school in which multiple infection waves occurred, i.e. representing the probability of new wave(s) of measles infections.

**Figure 2:**
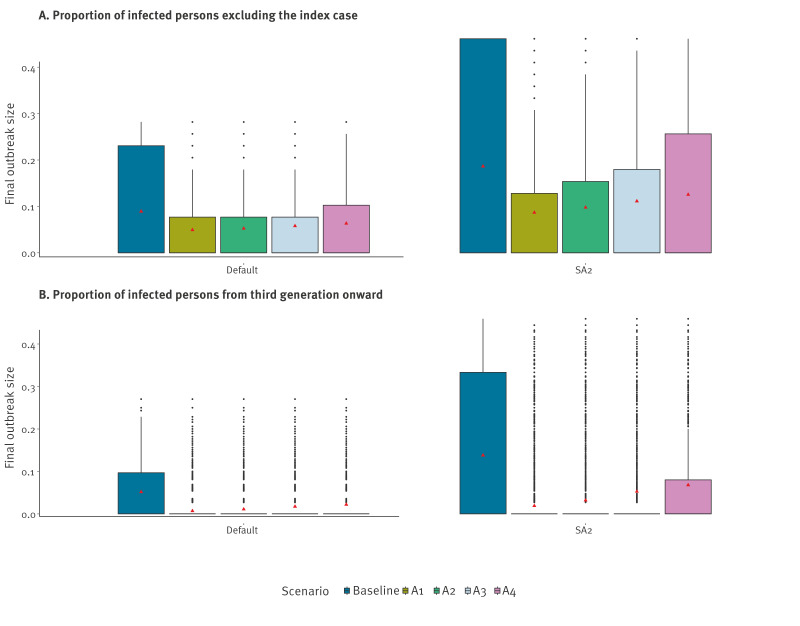
Simulation of the proportion of persons infected with measles virus in daycares, Flanders, Belgium

A1: 21 days quarantine; A2: 18 days quarantine; A3: quarantine from day 9 to day 21; A4: quarantine from day 9 to day 18); Baseline: no quarantine; SA2: sensitivity analysis with no immune infants (aged < 1 year) and 70% of toddlers (aged 1–3 years) were immune.

Quarantine of close contacts in primary schools and daycares could not prevent secondary infections, as exposure to and transmission from the index case had already occurred by the time the infected person was detected and isolated. This is illustrated by the average time between secondary case infection and detection of the index case, as made available in Supplementary Figure S2. However, by quarantining potential secondary cases before they become infectious, further infections can be avoided or reduced. Indeed, the average number of secondary cases caused by the index case was similar under all scenarios in each simulation (Table), indicating that reductions in onward transmission are primarily due to control measures affecting secondary cases. We found that in primary schools with a low immunity level of 20% and in daycares (Figure 2), the average final size of measles outbreaks could be substantially reduced (e.g. from 8.9% to 5.2% in daycares) when quarantine policies were in place, due to large reductions in the number of later-generation cases (e.g. from 5.2% to 1.1% in daycares) (Table). The full results for each scenario are available in Supplementary Tables S4–S9.

**Table:** Main results of the simulation of transmission of measles virus in primary schools and daycares, Flanders, Belgium

Setting	Proportion of non-index cases causing onward transmission	Probability of new wave(s)	Average number of secondary cases caused by index case	Proportion of non-index cases infectious after quarantine	Final proportion of later-generation cases
Panel A. Primary school					
Main analysis: ca 93% of children immune					
Baseline	0.113	0.125	0.732	NA	0.0005
A1	0.044	0.056	0.738	0.007	0.0002
A2	0.055	0.065	0.725	0.028	0.0002
Sensitivity analysis: 70% of children immune					
Baseline	0.168	0.383	2.206	NA	0.0039
A1	0.052	0.157	2.206	0.016	0.0014
A2	0.070	0.196	2.188	0.052	0.0019
Sensitivity analysis: 20% of children immune					
Baseline	0.137	0.552	5.651	NA	0.015
A1	0.042	0.249	5.676	0.020	0.006
A2	0.068	0.342	5.548	0.071	0.009
Panel B. Primary school, PEV provided					
Main analysis: ca 93% of children immune					
Baseline	0.078	0.088	0.740	NA	0.0003
A1	0.066	0.074	0.725	0.001	0.0003
A2	0.068	0.075	0.711	0.003	0.0003
Sensitivity analysis: 70% of children immune and no quarantine after PEV					
Baseline	0.118	0.281	2.223	NA	0.0024
A1	0.096	0.249	2.236	0.002	0.0022
A2	0.099	0.257	2.227	0.006	0.0023
Sensitivity analysis: 70% of children immune and quarantine after PEV					
Baseline	0.119	0.287	2.239	NA	0.0025
A1	0.048	0.152	2.221	0.010	0.0013
A2	0.058	0.168	2.215	0.039	0.0013
Sensitivity analysis: 20% of children immune and no quarantine after PEV					
Baseline	0.114	0.467	5.671	NA	0.009
A1	0.093	0.411	5.756	0.002	0.008
A2	0.098	0.417	5.611	0.009	0.009
Sensitivity analysis: 20% of children immune and quarantine after PEV					
Baseline	0.115	0.479	5.808	NA	0.009
A1	0.036	0.229	5.683	0.014	0.005
A2	0.051	0.281	5.646	0.054	0.007
Panel C. Daycare					
Main analysis: 10% of infants (< 1 years) immune, 89% of 1–3-year-olds immune					
Baseline	0.208	0.348	1.710	NA	0.052
A1	0.035	0.080	1.691	0.015	0.007
A2	0.054	0.120	1.676	0.049	0.011
Sensitivity analysis: no immune infants (< 1 years), 70% of 1–3-year-olds immune					
Baseline	0.216	0.432	2.771	NA	0.138
A1	0.035	0.108	2.798	0.017	0.019
A2	0.062	0.187	2.810	0.055	0.032

Baseline:  no quarantine; A1: 21 days quarantine; A2: 18 days quarantine; NA: not applicable; PEV: post-exposure vaccination.

Default assumptions:  10% of infants aged < 1 year were immune and 89% of 1–3-year-olds (i.e. main analysis). Red triangles indicate the mean across simulation runs.

Number of simulations: 10,000.

Quarantine policies effectively reduced the proportion of non-index cases causing onward transmission by 50–69% in primary schools and by 71–84% in daycares, as well as reducing the probability of subsequent infection waves by 38–59% in primary schools and by 57–77% in daycares, relative to the baseline scenario without quarantine (Table). Shortening the quarantine period to 18 days in primary schools and in daycares increased the proportion of infected contacts that remained infectious after the quarantine ended up to threefold, but the risk of a subsequent infection wave in the simulated populations was increased by only 16–37% in primary schools and 50–73% in daycares, relative to a quarantine of 21 days, and the final proportion of later-generation cases remained at most 3.2% of the school or daycare (Table). In addition, under all investigated policies in both primary school and daycare settings, at most 3.43% of infections were attributable to individuals returning from quarantine. The results from scenarios A3 and A4, where children were quarantined from day 9 onward, are presented in Supplementary Tables S4–S9. Apart from primary schools with an immunity level of ca 93%, this policy led to substantially more onward transmission. Additional sensitivity analyses in which we increased the reproduction number *R*_s_ or assumed a longer incubation period, or in which we varied assumptions about disease progression and contact rates, are presented in Supplementary Tables S4, S5 and S7, and resulted in similar conclusions. Results from simulations without heterogeneity in transmission (i.e. no overdispersion in the reproduction number) are presented in Supplementary Tables S10–S13 and led to the same conclusions, albeit with a higher risk of sustained transmission in general.

### Impact of post-exposure vaccination in primary school

**Figure 3:**
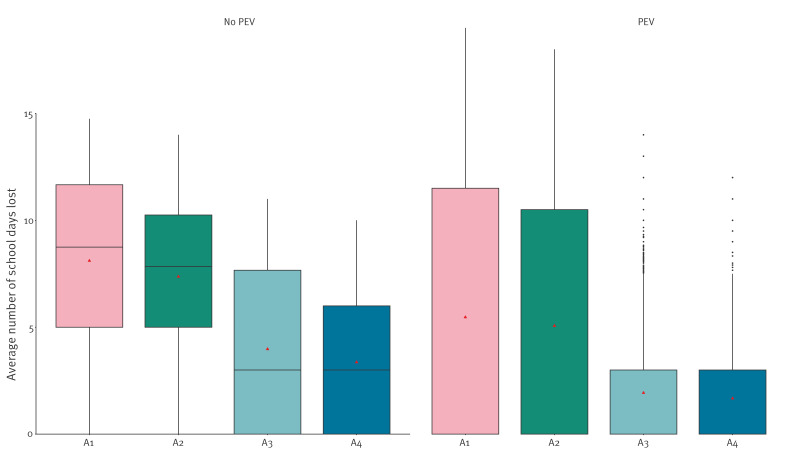
Average number of school days lost in a simulation of transmission of measles virus in primary schools, with 70% of primary school children being immune, Flanders, Belgium

A1: 21 days quarantine; A2: 18 days quarantine; A3: quarantine from day 9 to day 21; A4: quarantine from day 9 to day 18; PEV: post-exposure vaccination.

In the absence of quarantine of exposed susceptible contacts in primary schools with an immunity level of ca 93%, PEV alone reduced the probability of subsequent infection waves from 12.5% to 8.8% and resulted in similar risk compared with quarantining susceptible classmates for 18 days (probability of subsequent infection waves of 7%) (Table). Combining PEV with quarantine resulted in the highest reductions overall (Figure 1). For an immunity level of 70%, using PEV instead of quarantining susceptible classmates for 18 days increased the probability of subsequent infection waves from 19.6% to 25.7% (Table), while reducing the average number of school days lost by ca 30% since only those refusing PEV were quarantined (Figure 3). In schools with a low immunity level of 20%, PEV alone could not reduce transmission risk to an acceptable level but when combined with an 18-day quarantine it could further reduce the risk of future generations from 47.9% to 28.1% (Table). Results from a sensitivity analysis with a lower diagnosis rate are presented in Supplementary Table S8, and in such settings the impact of quarantine was lower, with the probability of subsequent infection waves reduced by only 24–33% relative to no quarantine. However, using PEV without quarantine in such settings resulted in a probability of subsequent infection waves that was only 5% increased relative to quarantine only. Another sensitivity analysis in which multiple introductions occurred is presented in Supplementary Table S9, showing that in such situations, the addition of PEV could result in reductions of up to 27% in the probability of subsequent infection waves relative to quarantine only.

## Discussion

Reducing contacts via isolation of cases and quarantine of exposed susceptible children is a well-established strategy to control measles outbreaks by limiting secondary transmission [10]. This study shows that the potential impact of quarantine policies is mainly defined by the proportion of immune individuals in the specific setting under consideration. It should be noted that we assumed a relatively high diagnosis rate in our main analyses, and in sensitivity analyses, in which children were assumed to be less likely to visit a doctor, the impact of quarantine was lower. Since some children are infectious before the start of the quarantine period and implementation is more challenging, quarantining children from day 9 onward is not recommended. In line with other modelling studies [10], we found that PEV alone does not have an additional benefit due to the delay between case detection and vaccination of susceptible classmates unless combined with quarantine or if implemented immediately upon detection of the index case, which is not feasible in practice. However, we found that in schools with a moderate immunity level of 70%, using PEV instead of an 18-day quarantine reduces the number of school days lost, and a trade-off could be considered between the risk of further spread and reducing educational/societal impact. In case of lower diagnosis rates, PEV may be a good alternative since it results in similar risk, even without quarantine, compared with an 18-day quarantine alone and has the additional advantage of creating future protection in uninfected classmates.

A real-world implementation of ‘ideal’ quarantine policies for measles comes with considerable logistical challenges and costs [12]. First, without high-quality immunisation information systems, it is difficult to quickly obtain estimates of the vaccination coverage in primary schools and daycares, or within subgroups like classrooms or groups of infants. Second, many resources are needed to verify compliance and monitor individuals for symptoms while in quarantine or isolation, and this is therefore often not performed in practice. Third, families may experience psychological distress, loss of income due to work absences, and primary school children may miss a considerable number of classes, especially for the relatively long quarantine periods typically put in place for measles [20]. We found that in settings with moderate-to-high vaccination coverage (e.g., above 70%), PEV even in the form of ‘reactive vaccination’ could be an alternative to quarantine, especially for lower diagnosis rates.

This simulation study has several limitations. First, we did not account for compliance with home quarantine or (secondary) vaccine failure. However, measles transmission from a person with prior immunity has rarely been documented [21]. Second, we assumed that all adults were immune due to vaccination or previous infection. Although it is generally accepted that natural immunity is lifelong, the proportion of the population that gained humoral immunity through widespread measles circulation and exposure is decreasing as a result of an ageing population, with population immunity shifting from predominantly natural immunity to solely vaccine-induced immunity, for which concerns about secondary vaccine failure have been raised [9,22]. Third, we assumed a single introduction of measles into the school or daycare. In reality, it is possible that multiple independent introductions occur, especially when measles circulation is high outside of the school environment. In a sensitivity analysis accounting for this, the probability of subsequent infection waves increased substantially in the absence of interventions but can be markedly reduced by quarantining exposed children, especially in combination with reactive vaccination since some children receiving PEV who were not infected will be immune by the time a new case is introduced. Fourth, although onward transmission after quarantine was limited in our simulations, this is likely in part due to depletion of the susceptible population, while the consequences of reducing the length of quarantine in terms of onward transmission are in reality not limited to the setting under consideration and further transmission can occur in other settings. Accounting for this is especially important in case of high clustering of unvaccinated individuals, which poses challenges to measles elimination efforts and can cause large outbreaks in otherwise highly vaccinated populations [23]. Measles, however, tends to spread in semi-closed settings such as primary schools during the early stages of an outbreak, and reducing transmission in schools and daycares is paramount to reduce the extent of an outbreak in the larger community [24]. Lastly, we did not directly validate our results against empirical measles outbreak data. While such validation would be valuable for further refining policy recommendations, this was considered beyond the scope of the present study. Instead, our model serves as a policy exploration framework that identifies robust patterns across a wide range of plausible epidemiological and behavioural scenarios. Future research could investigate calibration and validation of this framework against historical measles outbreak data to strengthen the empirical basis for specific policy recommendations. Importantly, while our findings have informed real-world decision-making in Flanders, they should not be looked at in isolation. The need for real-world experience remains essential for confirming and refining policy choices suggested by simulation-based analyses. In the long term, closer integration of epidemiological surveillance with modelling tools could support rapid scenario testing during outbreaks and more proportionate, evidence-informed decision-making. While this study deals specifically with measles, the use of a school transmission model to investigate viral spread has relevance to other infections. For example, these methods are applicable to other respiratory diseases, such as COVID-19, and can aid in preparedness for future health threats, in that they can be used to assess mitigation strategies for outbreaks in a school or daycare setting.

## Conclusion

Longer quarantine periods, aiming to span the maximal incubation period of 21 days, reduced the risk of onward transmission only marginally more than a shorter period of 18 days. This is important, as acceptability of and compliance with shorter quarantine periods might be higher. Findings from the present study have informed a revision of public health policy in Flanders to include a reduced quarantine period of 18 days after last exposure for susceptible children in primary schools with an immunity level below 70% and in daycares. While childhood vaccination remains the most cost-effective strategy for preventing measles outbreaks, quarantine policies serve as an important mitigation measure when immunity gaps persist.

## Data Availability

All analyses were performed using R software version 4.3.2. R code will be made available on Github upon publication (https://github.com/cecilekremer/measles_isolation).

## Supplementary Data

754000 Supplementary Material
