# Supplementary material for: Informing quarantine policy for measles control in primary school and daycare settings: insights from a simulation study, Flanders, Belgium
Source: Euro Surveill. 2026 Jun 4;31(22):2500763. doi: 10.2807/1560-7917.ES.2026.31.22.2500763 (PMC13241790; doi:10.2807/1560-7917.ES.2026.31.22.2500763)
Supplement: Supplementary Material [file 25-00763_KREMER_Supplement.pdf]

**Disclaimer:** This supplementary material is hosted by *Eurosurveillance* as supporting information alongside the article "Informing quarantine policy for measles control in primary school and daycare settings in Flanders, Belgium: insights from a simulation study", on behalf of the authors, who remain responsible for the accuracy and appropriateness of the content. The same standards for ethics, copyright, attributions and permissions as for the article apply. Supplements are not edited by *Eurosurveillance* and the journal is not responsible for the maintenance of any links or email addresses provided therein.

## Supplementary Tables

| **Parameter** | **Value / distribution** | Reference |
|---|---|---|
| Infectious period | 9 days (4 days before until 4 days after rash onset) | [1] |
| Proportion symptomatic children | 1 | assumed |
| Proportion symptomatic adults | 1 | assumed |
| Time from exposure to first symptoms | Lognormal(2.53; 0.21) | [2] |
| Time from first symptoms to rash onset | Uniform(3, 5) | [2] |
| Duration of isolation detected cases | $t_{rash} + 4$ | [1] |
| Probability of taking PCR test | 0.9 | assumed |
| PCR test sensitivity - >4 days before rash onset | 0.85 | [1] |
| PCR test sensitivity - $\leq$ 4 days before rash onset | 0.95 | [1] |
| Time from infection to detectable VL | 3 days | [1] |
| Proportion immune adults $p_A$ | 1 | assumed |
| Proportion immune children, school year 1-3 $p_{C_1}$ | 0.89 | [3] |
| Proportion immune children, school year 4-6 $p_{C_2}$ | 0.95 | assumed |
| Willingness to accept PEV | 0.90 | assumed |
| Efficacy of PEV | 0.85 | [4] |
| School size | 420 | assumed |
| Contact frequency within class $\lambda_w$ | class size - 1 | assumed |
| Contact frequency between classes $\lambda_b$ | 2.5 | [5] |
| Basic reproduction number $R_s$ | 12 | [6] |
| Overdispersion parameter $k$ | 0.3 | [7] |
| Contact rate $c$ | $\lambda_w + \lambda_b$ | |

Table S1: Model parameters for the school setting.

| Parameter | Value / distribution | Reference |
|---|---|---|
| Infectious period | 9 days (4 days before until 4 days after rash onset) | [1] |
| Proportion symptomatic children | 1 | assumed |
| Proportion symptomatic adults | 1 | assumed |
| Time from exposure to first symptoms | Lognormal(2.53; 0.21) | [2] |
| Time from first symptoms to rash onset | Uniform(3, 5) | [2] |
| Duration of isolation detected cases | $t_{rash} + 4$ | [1] |
| Probability of taking PCR test | 0.9 | assumed |
| PCR test sensitivity - >4 days before rash onset | 0.85 | [1] |
| PCR test sensitivity - $\leq$ 4 days before rash onset | 0.95 | [1] |
| Time from infection to detectable VL | 3 days | [1] |
| Proportion immune adults $p_A$ | 1 | assumed |
| Proportion immune children, under 1 year $p_{C_1}$ | 0.1 | [8] |
| Proportion immune children, 1-3 years $p_{C_2}$ | 0.89 | [3] |
| Daycare size (daily) | 21 | 3 adults, 18 children |
| Daycare pool size | 36 | total number of children |
| Contact rate/frequency $c/\lambda_w$ | daycare size - 1 | assumed |
| Basic reproduction number $R_s$ | 12 | [6] |
| Overdispersion parameter $k$ | 0.3 | [7] |

Table S2: Model parameters for the daycare setting.

| Outcome | Description |
|---|---|
| $FS_{\text{all}}$ | Final size, i.e., proportion of the school / daycare that got infected (excluding index case) |
| $FS_{\text{newWave}}$ | Final size excluding index and its secondary cases, i.e., proportion of the school / daycare that got infected by non-index cases |
| $R_{\text{index}}$ | Number of secondary cases caused by the index case |
| $P_{\text{onward}}$ | Proportion of non-index cases causing onward transmission |
| $P_{\text{newWave}}$ | Proportion of simulation runs that have at least a ‘second wave’, i.e. where there is onward transmission from non-index cases |
| $P_{\text{infQ}}$ | Proportion of non-index cases that are still infectious after being quarantined as a close contact, i.e., the potential for onward transmission |
| $P_{\text{onwardQ}}$ | Proportion of infections that are attributable to quarantined (non-index) cases after they return to school / daycare height |

Table S3: Outbreak characteristics to investigate the impact of different quarantine policies.

| **Scenario** | $FS_{\text{all}}$ | $FS_{\text{newWave}}$ | $R_{\text{index}}$ | $P_{\text{onward}}$ | $P_{\text{infQ}}$ | $P_{\text{onwardQ}}$ | $P_{\text{newWave}}$ |
|---|---|---|---|---|---|---|---|
| Baseline | 0.0020 | 0.0005 | 0.7322 | 0.1126 | n.a. | n.a. | 0.1249 |
| A1 | 0.0017 | 0.0002 | 0.7375 | 0.0439 | 0.0074 | 0.0009 | 0.0555 |
| A2 | 0.0017 | 0.0002 | 0.7246 | 0.0550 | 0.0280 | 0.0045 | 0.0649 |
| A3 | 0.0018 | 0.0003 | 0.7345 | 0.0583 | 0.0097 | 0.0012 | 0.0694 |
| A4 | 0.0018 | 0.0003 | 0.7196 | 0.0689 | 0.0255 | 0.0041 | 0.0781 |
| *Sensitivity analysis 1:* $R_0 = 18$ | | | | | | | |
| Baseline | 0.0023 | 0.0006 | 0.8343 | 0.1184 | n.a. | n.a. | 0.1428 |
| A1 | 0.0020 | 0.0003 | 0.8259 | 0.0530 | 0.0093 | 0.0013 | 0.0723 |
| A2 | 0.0020 | 0.0003 | 0.8064 | 0.0597 | 0.0302 | 0.0045 | 0.0780 |
| A3 | 0.0021 | 0.0004 | 0.8297 | 0.0670 | 0.0097 | 0.0019 | 0.0905 |
| A4 | 0.0020 | 0.0004 | 0.8096 | 0.0736 | 0.0298 | 0.0048 | 0.0953 |
| *Sensitivity analysis 2: 70% of children immune* | | | | | | | |
| Baseline | 0.0084 | 0.0039 | 2.2061 | 0.1680 | n.a. | n.a. | 0.3834 |
| A1 | 0.0059 | 0.0014 | 2.2059 | 0.0523 | 0.0155 | 0.0038 | 0.1574 |
| A2 | 0.0063 | 0.0019 | 2.1875 | 0.0699 | 0.0516 | 0.0125 | 0.1957 |
| A3 | 0.0066 | 0.0021 | 2.1996 | 0.0936 | 0.0208 | 0.0060 | 0.2377 |
| A4 | 0.0071 | 0.0026 | 2.2146 | 0.1096 | 0.0509 | 0.0148 | 0.2739 |
| *Sensitivity analysis 3: 20% of children immune* | | | | | | | |
| Baseline | 0.0259 | 0.0146 | 5.6511 | 0.1371 | n.a. | n.a. | 0.5515 |
| A1 | 0.0171 | 0.0056 | 5.6758 | 0.0417 | 0.0202 | 0.0063 | 0.2490 |
| A2 | 0.0199 | 0.0087 | 5.5481 | 0.0680 | 0.0711 | 0.0208 | 0.3421 |
| A3 | 0.0220 | 0.0105 | 5.7118 | 0.0983 | 0.0292 | 0.0119 | 0.4344 |
| A4 | 0.0234 | 0.0121 | 5.6666 | 0.1135 | 0.0548 | 0.0209 | 0.4707 |
| *Sensitivity analysis 9: longer incubation period (median 14 days)* | | | | | | | |
| Baseline | 0.0019 | 0.0005 | 0.7197 | 0.1086 | n.a. | n.a. | 0.1155 |
| A1 | 0.0018 | 0.0002 | 0.7488 | 0.0482 | 0.0163 | 0.0023 | 0.0592 |
| A2 | 0.0017 | 0.0003 | 0.7157 | 0.0600 | 0.0431 | 0.0068 | 0.0695 |
| A3 | 0.0017 | 0.0002 | 0.7288 | 0.0537 | 0.0162 | 0.0021 | 0.0641 |
| A4 | 0.0018 | 0.0003 | 0.7161 | 0.0710 | 0.0454 | 0.0071 | 0.0802 |

Table S4: Simulation results for the school setting. Baseline = no quarantine, A1 = 21 days quarantine, A2 = 18 days quarantine, A3 = quarantine from day 9 to day 21, A4 = quarantine from day 9 to day 18.

| Scenario | $FS_{\text{all}}$ | $FS_{\text{newWave}}$ | $R_{\text{index}}$ | $P_{\text{onward}}$ | $P_{\text{infQ}}$ | $P_{\text{onwardQ}}$ | $P_{\text{newWave}}$ |
|---|---|---|---|---|---|---|---|
| *Sensitivity analysis 4: peaked infectiousness profile $i(t)$* | | | | | | | |
| Baseline | 0.0004 | 0 | 0.1725 | 0.0591 | n.a. | n.a. | 0.0185 |
| A1 | 0.0004 | 0 | 0.1711 | 0.0197 | 0.0030 | 0.0002 | 0.0062 |
| A2 | 0.0004 | 0 | 0.1776 | 0.0181 | 0.0092 | 0.0005 | 0.0063 |
| A3 | 0.0004 | 0 | 0.1726 | 0.0239 | 0.0035 | 0.0001 | 0.0077 |
| A4 | 0.0004 | 0 | 0.1768 | 0.0283 | 0.0103 | 0.0009 | 0.0092 |
| *Sensitivity analysis 5: detection at time of symptom onset* | | | | | | | |
| Baseline | 0.0019 | 0.0004 | 0.7213 | 0.1072 | n.a. | n.a. | 0.1146 |
| A1 | 0.0017 | 0.0002 | 0.7226 | 0.0463 | 0.0092 | 0.0011 | 0.0566 |
| A2 | 0.0018 | 0.0003 | 0.7300 | 0.0563 | 0.0285 | 0.0037 | 0.0672 |
| A3 | 0.0018 | 0.0003 | 0.7303 | 0.0630 | 0.0088 | 0.0011 | 0.0745 |
| A4 | 0.0018 | 0.0003 | 0.7176 | 0.0673 | 0.0262 | 0.0043 | 0.0772 |
| *Sensitivity analysis 6: higher probability of detection closer to rash onset and no later than rash onset* | | | | | | | |
| Baseline | 0.0019 | 0.0005 | 0.7259 | 0.1049 | n.a. | n.a. | 0.1146 |
| A1 | 0.0017 | 0.0002 | 0.7233 | 0.0457 | 0.0078 | 0.0010 | 0.0546 |
| A2 | 0.0017 | 0.0003 | 0.7224 | 0.0541 | 0.0257 | 0.0035 | 0.0657 |
| A3 | 0.0018 | 0.0003 | 0.7171 | 0.0649 | 0.0090 | 0.0013 | 0.0750 |
| A4 | 0.0018 | 0.0003 | 0.7242 | 0.0641 | 0.0269 | 0.0039 | 0.0740 |
| *Sensitivity analysis 7: higher between-class contact rate ($\lambda_b = 6$)* | | | | | | | |
| Baseline | 0.0024 | 0.0007 | 0.8060 | 0.1332 | n.a. | n.a. | 0.1559 |
| A1 | 0.0020 | 0.0004 | 0.7891 | 0.0734 | 0.0081 | 0.0011 | 0.0952 |
| A2 | 0.0020 | 0.0004 | 0.7822 | 0.0812 | 0.0266 | 0.0050 | 0.1005 |
| A3 | 0.0021 | 0.0005 | 0.8111 | 0.0837 | 0.0081 | 0.0014 | 0.1083 |
| A4 | 0.0021 | 0.0005 | 0.8062 | 0.0923 | 0.0267 | 0.0046 | 0.1156 |
| *Sensitivity analysis 8: susceptible contacts only isolated once* | | | | | | | |
| Baseline | 0.0020 | 0.0005 | 0.7289 | 0.1080 | n.a. | n.a. | 0.1150 |
| A1 | 0.0017 | 0.0002 | 0.7195 | 0.0465 | 0.0092 | 0.0010 | 0.0561 |
| A2 | 0.0018 | 0.0003 | 0.7434 | 0.0515 | 0.0260 | 0.0033 | 0.0641 |
| A3 | 0.0017 | 0.0003 | 0.7090 | 0.0629 | 0.0095 | 0.0010 | 0.0737 |
| A4 | 0.0018 | 0.0003 | 0.7326 | 0.0686 | 0.0271 | 0.0047 | 0.0818 |

Table S5: Simulation results for the school setting - additional sensitivity analyses. Baseline = no quarantine, A1 = 21 days quarantine, A2 = 18 days quarantine, A3 = quarantine from day 9 to day 21, A4 = quarantine from day 9 to day 18

| Scenario | $FS_{\text{all}}$ | $FS_{\text{newWave}}$ | $R_{\text{index}}$ | $P_{\text{onward}}$ | $P_{\text{infQ}}$ | $P_{\text{onwardQ}}$ | $P_{\text{newWave}}$ |
|---|---|---|---|---|---|---|---|
| Baseline | 0.0018 | 0.0003 | 0.7401 | 0.0775 | n.a. | n.a. | 0.0878 |
| A1 | 0.0018 | 0.0003 | 0.7254 | 0.0660 | 0.0011 | 0.0001 | 0.0738 |
| A2 | 0.0017 | 0.0003 | 0.7111 | 0.0683 | 0.0032 | 0.0003 | 0.0751 |
| A3 | 0.0017 | 0.0003 | 0.7038 | 0.0686 | 0.0010 | 0.0000 | 0.0757 |
| A4 | 0.0018 | 0.0003 | 0.7233 | 0.0704 | 0.0031 | 0.0003 | 0.0807 |
| *Sensitivity analysis 1: quarantine even after PEV* | | | | | | | |
| Baseline | 0.0018 | 0.0003 | 0.7073 | 0.0806 | n.a. | n.a. | 0.0877 |
| A1 | 0.0017 | 0.0002 | 0.7185 | 0.0473 | 0.0055 | 0.0003 | 0.0582 |
| A2 | 0.0017 | 0.0002 | 0.7283 | 0.0542 | 0.0217 | 0.0021 | 0.0652 |
| A3 | 0.0017 | 0.0002 | 0.7135 | 0.0558 | 0.0463 | 0.0048 | 0.0636 |
| A4 | 0.0018 | 0.0003 | 0.7166 | 0.0717 | 0.0736 | 0.0096 | 0.0791 |
| *Sensitivity analysis 2: 20% of children immune, no quarantine after PEV* | | | | | | | |
| Baseline | 0.0204 | 0.0090 | 5.6713 | 0.1137 | n.a. | n.a. | 0.4671 |
| A1 | 0.0199 | 0.0083 | 5.7564 | 0.0930 | 0.0024 | 0.0004 | 0.4108 |
| A2 | 0.0198 | 0.0085 | 5.6113 | 0.0978 | 0.0090 | 0.0015 | 0.4169 |
| A3 | 0.0200 | 0.0086 | 5.6208 | 0.1025 | 0.0044 | 0.0011 | 0.4322 |
| A4 | 0.0203 | 0.0090 | 5.6522 | 0.1044 | 0.0074 | 0.0017 | 0.4330 |
| *Sensitivity analysis 3: 20% of children immune, quarantine even after PEV* | | | | | | | |
| Baseline | 0.0209 | 0.0092 | 5.8076 | 0.1152 | n.a. | n.a. | 0.4792 |
| A1 | 0.0167 | 0.0052 | 5.6827 | 0.0360 | 0.0139 | 0.0026 | 0.2293 |
| A2 | 0.0179 | 0.0065 | 5.6463 | 0.0506 | 0.0543 | 0.0106 | 0.2806 |
| A3 | 0.0208 | 0.0092 | 5.7572 | 0.0700 | 0.1194 | 0.0186 | 0.3500 |
| A4 | 0.0248 | 0.0134 | 5.7188 | 0.1022 | 0.1858 | 0.0343 | 0.4367 |
| *Sensitivity analysis 4: 70% of children immune, no quarantine after PEV* | | | | | | | |
| Baseline | 0.0070 | 0.0024 | 2.2231 | 0.1175 | n.a. | n.a. | 0.2808 |
| A1 | 0.0068 | 0.0022 | 2.2360 | 0.0959 | 0.0016 | 0.0002 | 0.2494 |
| A2 | 0.0068 | 0.0023 | 2.2267 | 0.0989 | 0.0061 | 0.0007 | 0.2565 |
| A3 | 0.0067 | 0.0022 | 2.2182 | 0.0996 | 0.0027 | 0.0004 | 0.2516 |
| A4 | 0.0069 | 0.0023 | 2.2341 | 0.1036 | 0.0054 | 8e-04 | 0.2582 |
| *Sensitivity analysis 5: 70% of children immune, quarantine even after PEV* | | | | | | | |
| Baseline | 0.0070 | 0.0025 | 2.2289 | 0.1189 | n.a. | n.a. | 0.2873 |
| A1 | 0.0060 | 0.0013 | 2.2791 | 0.0476 | 0.0102 | 0.0012 | 0.1524 |
| A2 | 0.0060 | 0.0015 | 2.1930 | 0.0580 | 0.0394 | 0.0052 | 0.1678 |
| A3 | 0.0066 | 0.0020 | 2.2491 | 0.0759 | 0.0888 | 0.0127 | 0.2083 |
| A4 | 0.0073 | 0.0027 | 2.2779 | 0.1021 | 0.1452 | 0.0255 | 0.2562 |

Table S6: Simulation results for the school setting, including post-exposure vaccination (PEV). Baseline = no quarantine, A1 = 21 days quarantine, A2 = 18 days quarantine, A3 = quarantine from day 9 to day 21, A4 = quarantine from day 9 to day 18.

| Scenario | $FS_{\textbf{all}}$ | $FS_{\textbf{newWave}}$ | $R_{\textbf{index}}$ | $P_{\textbf{onward}}$ | $P_{\textbf{infQ}}$ | $P_{\textbf{onwardQ}}$ | $P_{\textbf{newWave}}$ |
|---|---|---|---|---|---|---|---|
| Baseline | 0.0892 | 0.0515 | 1.7099 | 0.2082 | 0.0000 | 0.0000 | 0.3477 |
| A1 | 0.0493 | 0.0068 | 1.6911 | 0.0351 | 0.0145 | 0.0027 | 0.0795 |
| A2 | 0.0524 | 0.0108 | 1.6760 | 0.0543 | 0.0494 | 0.0115 | 0.1201 |
| A3 | 0.0578 | 0.0172 | 1.6731 | 0.0807 | 0.0192 | 0.0051 | 0.1616 |
| A4 | 0.0632 | 0.0220 | 1.7221 | 0.1024 | 0.0457 | 0.0133 | 0.2011 |
| *Sensitivity analysis 1:* $R_0 = 18$ | | | | | | | |
| Baseline | 0.1043 | 0.0622 | 1.9357 | 0.2217 | 0.0000 | 0.0000 | 0.3982 |
| A1 | 0.0577 | 0.0086 | 1.9642 | 0.0349 | 0.0168 | 0.0037 | 0.0910 |
| A2 | 0.0628 | 0.0143 | 1.9648 | 0.0589 | 0.0552 | 0.0135 | 0.1468 |
| A3 | 0.0685 | 0.0221 | 1.9279 | 0.0914 | 0.0211 | 0.0067 | 0.1967 |
| A4 | 0.0721 | 0.0269 | 1.9023 | 0.1125 | 0.0504 | 0.0157 | 0.2378 |
| *Sensitivity analysis 2: no immune infants, 70% of 1-3yo immune* | | | | | | | |
| Baseline | 0.1864 | 0.1379 | 2.7712 | 0.2157 | 0.0000 | 0.0000 | 0.4316 |
| A1 | 0.0867 | 0.0189 | 2.7977 | 0.0350 | 0.0165 | 0.0039 | 0.1080 |
| A2 | 0.0977 | 0.0318 | 2.8100 | 0.0615 | 0.0594 | 0.0154 | 0.1870 |
| A3 | 0.1113 | 0.0526 | 2.6912 | 0.0969 | 0.0223 | 0.0073 | 0.2333 |
| A4 | 0.1254 | 0.0679 | 2.7479 | 0.1272 | 0.0490 | 0.0174 | 0.2912 |
| *Sensitivity analysis 3: longer incubation period (median 14 days)* | | | | | | | |
| Baseline | 0.0908 | 0.0523 | 1.7454 | 0.2069 | 0.0000 | 0.0000 | 0.3545 |
| A1 | 0.0521 | 0.0080 | 1.7617 | 0.0393 | 0.0290 | 0.0057 | 0.0899 |
| A2 | 0.0567 | 0.0148 | 1.7071 | 0.0731 | 0.0769 | 0.0201 | 0.1575 |
| A3 | 0.0573 | 0.0144 | 1.7525 | 0.0677 | 0.0298 | 0.0077 | 0.1417 |
| A4 | 0.0627 | 0.0208 | 1.7399 | 0.0976 | 0.0753 | 0.0210 | 0.1997 |

Table S7: Simulation results for the daycare setting. Baseline = no quarantine, A1 = 21 days quarantine, A2 = 18 days quarantine, A3 = quarantine from day 9 to day 21, A4 = quarantine from day 9 to day 18.

| Scenario | $FS_{\textbf{all}}$ | $FS_{\textbf{newWave}}$ | $R_{\textbf{index}}$ | $P_{\textbf{onward}}$ | $P_{\textbf{infQ}}$ | $P_{\textbf{onwardQ}}$ | $P_{\textbf{newWave}}$ |
|---|---|---|---|---|---|---|---|
| *70% of children immune - quarantine only* | | | | | | | |
| Baseline | 0.0106 | 0.0054 | 2.5619 | 0.1680 | n.a. | n.a. | 0.4202 |
| A1 | 0.0081 | 0.0030 | 2.5417 | 0.0972 | 0.0217 | 0.0052 | 0.2797 |
| A2 | 0.0089 | 0.0037 | 2.5783 | 0.1152 | 0.0618 | 0.0128 | 0.3190 |
| A3 | 0.0090 | 0.0039 | 2.5341 | 0.1263 | 0.0265 | 0.0061 | 0.3351 |
| A4 | 0.0095 | 0.0044 | 2.5283 | 0.1396 | 0.0609 | 0.0142 | 0.3622 |
| *70% of children immune - PEV with quarantine* | | | | | | | |
| Baseline | 0.0094 | 0.0042 | 2.5414 | 0.1374 | n.a. | n.a. | 0.3575 |
| A1 | 0.0082 | 0.0030 | 2.5565 | 0.0933 | 0.0158 | 0.0018 | 0.2731 |
| A2 | 0.0084 | 0.0032 | 2.5520 | 0.1030 | 0.0512 | 0.0068 | 0.2928 |
| A3 | 0.0090 | 0.0038 | 2.5540 | 0.1154 | 0.0890 | 0.0110 | 0.3149 |
| A4 | 0.0100 | 0.0047 | 2.5863 | 0.1386 | 0.1196 | 0.0206 | 0.3616 |
| *70% of children immune - PEV without quarantine* | | | | | | | |
| Baseline | 0.0096 | 0.0044 | 2.5863 | 0.1373 | n.a. | n.a. | 0.3614 |
| A1 | 0.0092 | 0.0039 | 2.6036 | 0.1217 | 0.0022 | 0.0002 | 0.3323 |
| A2 | 0.0090 | 0.0039 | 2.5377 | 0.1266 | 0.0074 | 0.0008 | 0.3345 |
| A3 | 0.0091 | 0.0039 | 2.5438 | 0.1252 | 0.0031 | 0.0005 | 0.3339 |
| A4 | 0.0094 | 0.0042 | 2.5307 | 0.1307 | 0.0072 | 0.0010 | 0.3438 |

Table S8: Simulation results for the school setting - additional sensitivity analyses with a lower diagnosis rate, i.e., a probability of getting tested of 50%. Baseline = no quarantine, A1 = 21 days quarantine, A2 = 18 days quarantine, A3 = quarantine from day 9 to day 21, A4 = quarantine from day 9 to day 18.

| **Scenario** | $FS_{\textbf{all}}$ | $FS_{\textbf{newWave}}$ | $R_{\textbf{index}}$ | $P_{\textbf{onward}}$ | $P_{\textbf{infQ}}$ | $P_{\textbf{onwardQ}}$ | $P_{\textbf{newWave}}$ |
|---|---|---|---|---|---|---|---|
| *70% of children immune - 3 introductions at time 0 - quarantine only* | | | | | | | |
| Baseline | 0.0185 | 0.0145 | 2.0137 | 0.1528 | n.a. | n.a. | 0.6746 |
| A1 | 0.0136 | 0.0096 | 1.9850 | 0.0324 | 0.0231 | 0.0063 | 0.1999 |
| A2 | 0.0145 | 0.0105 | 1.9845 | 0.0536 | 0.0803 | 0.0236 | 0.3046 |
| A3 | 0.0151 | 0.0111 | 1.9990 | 0.0759 | 0.0321 | 0.0111 | 0.3998 |
| A4 | 0.0158 | 0.0118 | 1.9755 | 0.0933 | 0.0710 | 0.0249 | 0.4634 |
| *70% of children immune - 3 introductions at time 0 - PEV with quarantine* | | | | | | | |
| Baseline | 0.0148 | 0.0109 | 1.9866 | 0.0870 | n.a. | n.a. | 0.4358 |
| A1 | 0.0134 | 0.0094 | 1.9902 | 0.0283 | 0.0166 | 0.0030 | 0.1767 |
| A2 | 0.0138 | 0.0098 | 1.9849 | 0.0377 | 0.0581 | 0.0099 | 0.2227 |
| A3 | 0.0148 | 0.0108 | 2.0131 | 0.0573 | 0.1296 | 0.0218 | 0.3078 |
| A4 | 0.0159 | 0.0119 | 1.9889 | 0.0835 | 0.2041 | 0.0407 | 0.4055 |
| *70% of children immune - 3 introductions at time 0 - PEV without quarantine* | | | | | | | |
| Baseline | 0.0149 | 0.0109 | 1.9880 | 0.0854 | n.a. | n.a. | 0.4325 |
| A1 | 0.0145 | 0.0104 | 1.9911 | 0.0656 | 0.0026 | 0.0004 | 0.3460 |
| A2 | 0.0146 | 0.0105 | 1.9883 | 0.0685 | 0.0088 | 0.0011 | 0.3579 |
| A3 | 0.0146 | 0.0106 | 1.9985 | 0.0692 | 0.0036 | 0.0006 | 0.3626 |
| A4 | 0.0147 | 0.0107 | 2.0020 | 0.0742 | 0.0079 | 0.0017 | 0.3816 |
| *70% of children immune - one introduction every 10 days - quarantine only* | | | | | | | |
| Baseline | 0.1689 | 0.1670 | 1.2749 | 0.1364 | n.a. | n.a. | 1.0000 |
| A1 | 0.1445 | 0.1428 | 1.0890 | 0.0297 | 0.0273 | 0.0073 | 0.8508 |
| A2 | 0.1479 | 0.1461 | 1.1046 | 0.0489 | 0.0792 | 0.0248 | 0.9573 |
| A3 | 0.1517 | 0.1499 | 1.1349 | 0.0699 | 0.0353 | 0.0122 | 0.9909 |
| A4 | 0.1552 | 0.1533 | 1.1591 | 0.0850 | 0.0732 | 0.0265 | 0.9983 |
| *70% of children immune - one introduction every 10 days - PEV with quarantine* | | | | | | | |
| Baseline | 0.0990 | 0.0967 | 1.2736 | 0.0616 | n.a. | n.a. | 0.9421 |
| A1 | 0.0941 | 0.0920 | 1.1995 | 0.0234 | 0.0180 | 0.0021 | 0.6126 |
| A2 | 0.0949 | 0.0927 | 1.2044 | 0.0313 | 0.0588 | 0.0080 | 0.7188 |
| A3 | 0.0961 | 0.0940 | 1.2120 | 0.0402 | 0.1239 | 0.0139 | 0.7963 |
| A4 | 0.0972 | 0.0951 | 1.2067 | 0.0597 | 0.1971 | 0.0292 | 0.9040 |
| *70% of children immune - one introduction every 10 days - PEV without quarantine* | | | | | | | |
| Baseline | 0.0989 | 0.0967 | 1.2714 | 0.0625 | n.a. | n.a. | 0.9472 |
| A1 | 0.0963 | 0.0940 | 1.2253 | 0.0429 | 0.0041 | 0.0003 | 0.8403 |
| A2 | 0.0966 | 0.0944 | 1.2291 | 0.0456 | 0.0105 | 0.0011 | 0.8595 |
| A3 | 0.0971 | 0.0949 | 1.2394 | 0.0486 | 0.0054 | 0.0007 | 0.8777 |
| A4 | 0.0976 | 0.0954 | 1.2450 | 0.0511 | 0.0103 | 0.0013 | 0.8945 |

Table S9: Simulation results for the school setting - additional sensitivity analyses with multiple introductions. Baseline = no quarantine, A1 = 21 days quarantine, A2 = 18 days quarantine, A3 = quarantine from day 9 to day 21, A4 = quarantine from day 9 to day 18.

| Scenario | $FS_{\text{all}}$ | $FS_{\text{newWave}}$ | $R_{\text{index}}$ | $P_{\text{onward}}$ | $P_{\text{infQ}}$ | $P_{\text{onwardQ}}$ | $P_{\text{newWave}}$ |
|---|---|---|---|---|---|---|---|
| Baseline | 0.0046 | 0.0017 | 1.3839 | 0.1858 | n.a. | n.a. | 0.3472 |
| A1 | 0.0036 | 0.0008 | 1.3872 | 0.0800 | 0.0163 | 0.0035 | 0.1757 |
| A2 | 0.0037 | 0.0009 | 1.3762 | 0.0923 | 0.0546 | 0.0132 | 0.1970 |
| A3 | 0.0038 | 0.0010 | 1.3655 | 0.1040 | 0.0199 | 0.0051 | 0.2159 |
| A4 | 0.0039 | 0.0011 | 1.3687 | 0.1184 | 0.0513 | 0.0127 | 0.2396 |
| *Sensitivity analysis 1:* $R_0 = 18$ | | | | | | | |
| Baseline | 0.0056 | 0.0023 | 1.6260 | 0.1803 | n.a. | n.a. | 0.3842 |
| A1 | 0.0045 | 0.0012 | 1.6067 | 0.0947 | 0.0162 | 0.0044 | 0.2380 |
| A2 | 0.0046 | 0.0013 | 1.6110 | 0.1062 | 0.0591 | 0.0139 | 0.2538 |
| A3 | 0.0047 | 0.0014 | 1.6197 | 0.1159 | 0.0194 | 0.0049 | 0.2755 |
| A4 | 0.0049 | 0.0016 | 1.6338 | 0.1330 | 0.0575 | 0.0145 | 0.3037 |
| *Sensitivity analysis 2: 70% of children immune* | | | | | | | |
| Baseline | 0.0221 | 0.0134 | 4.3218 | 0.2286 | n.a. | n.a. | 0.8302 |
| A1 | 0.0139 | 0.0053 | 4.2540 | 0.0752 | 0.0278 | 0.0119 | 0.4154 |
| A2 | 0.0162 | 0.0075 | 4.2826 | 0.1076 | 0.0939 | 0.0384 | 0.5046 |
| A3 | 0.0171 | 0.0085 | 4.2704 | 0.1417 | 0.0407 | 0.0202 | 0.5963 |
| A4 | 0.0191 | 0.0105 | 4.2554 | 0.1701 | 0.0859 | 0.0392 | 0.6659 |
| *Sensitivity analysis 3: 20% of children immune* | | | | | | | |
| Baseline | 0.0525 | 0.0313 | 10.7686 | 0.1520 | n.a. | n.a. | 0.9396 |
| A1 | 0.0428 | 0.0212 | 10.8220 | 0.0574 | 0.0310 | 0.0191 | 0.5753 |
| A2 | 0.0669 | 0.0459 | 10.8276 | 0.1084 | 0.1143 | 0.0623 | 0.7815 |
| A3 | 0.0577 | 0.0364 | 10.8966 | 0.1377 | 0.0505 | 0.0270 | 0.8687 |
| A4 | 0.0624 | 0.0412 | 10.8698 | 0.1533 | 0.0783 | 0.0411 | 0.9162 |
| *Sensitivity analysis 9: longer incubation period (median 14 days)* | | | | | | | |
| Baseline | 0.0046 | 0.0017 | 1.4060 | 0.1818 | 0.0000 | 0.0000 | 0.3426 |
| A1 | 0.0037 | 0.0008 | 1.4009 | 0.0873 | 0.0305 | 0.0070 | 0.1913 |
| A2 | 0.0039 | 0.0011 | 1.3767 | 0.1087 | 0.0871 | 0.0216 | 0.2253 |
| A3 | 0.0038 | 0.0010 | 1.3892 | 0.0989 | 0.0310 | 0.0083 | 0.2119 |
| A4 | 0.0040 | 0.0012 | 1.3970 | 0.1219 | 0.0858 | 0.0235 | 0.2498 |

Table S10: Simulation results for the school setting - **no heterogeneity in transmission**. Baseline = no quarantine, A1 = 21 days quarantine, A2 = 18 days quarantine, A3 = quarantine from day 9 to day 21, A4 = quarantine from day 9 to day 18.

| Scenario | $FS_{\text{all}}$ | $FS_{\text{newWave}}$ | $R_{\text{index}}$ | $P_{\text{onward}}$ | $P_{\text{infQ}}$ | $P_{\text{onwardQ}}$ | $P_{\text{newWave}}$ |
|---|---|---|---|---|---|---|---|
| *Sensitivity analysis 4: peaked infectiousness profile $i(t)$* | | | | | | | |
| Baseline | 8e-04 | 0.0002 | 0.3157 | 0.1047 | n.a. | n.a. | 0.0593 |
| A1 | 0.0007 | 0.0001 | 0.3047 | 0.0383 | 0.0056 | 0.0007 | 0.0220 |
| A2 | 0.0007 | 0.0001 | 0.3038 | 0.0469 | 0.0183 | 0.0026 | 0.0264 |
| A3 | 0.0007 | 0.0001 | 0.3150 | 0.0440 | 0.0072 | 0.0009 | 0.0261 |
| A4 | 0.0007 | 0.0001 | 0.3054 | 0.0500 | 0.0179 | 0.0023 | 0.0285 |
| *Sensitivity analysis 5: detection at time of symptom onset* | | | | | | | |
| Baseline | 0.0046 | 0.0018 | 1.3881 | 0.1906 | n.a. | n.a. | 0.3572 |
| A1 | 0.0036 | 0.0008 | 1.3807 | 0.0799 | 0.0152 | 0.0032 | 0.1765 |
| A2 | 0.0037 | 0.0009 | 1.3871 | 0.0950 | 0.0543 | 0.0126 | 0.2053 |
| A3 | 0.0038 | 0.0010 | 1.3611 | 0.1042 | 0.0197 | 0.0049 | 0.2181 |
| A4 | 0.0040 | 0.0011 | 1.4000 | 0.1189 | 0.0542 | 0.0139 | 0.2459 |
| *Sensitivity analysis 6: higher probability of detection closer to rash onset and no later than rash onset* | | | | | | | |
| Baseline | 0.0045 | 0.0018 | 1.3584 | 0.1906 | n.a. | n.a. | 0.3495 |
| A1 | 0.0036 | 0.0008 | 1.3774 | 0.0820 | 0.0154 | 0.0038 | 0.1822 |
| A2 | 0.0037 | 0.0009 | 1.3677 | 0.0918 | 0.0529 | 0.0128 | 0.1942 |
| A3 | 0.0038 | 0.0010 | 1.3836 | 0.1075 | 0.0190 | 0.0054 | 0.2251 |
| A4 | 0.0040 | 0.0011 | 1.4077 | 0.1164 | 0.0531 | 0.0128 | 0.2413 |
| *Sensitivity analysis 7: higher between-class contact rate ($\lambda_b = 6$)* | | | | | | | |
| Baseline | 0.0061 | 0.0030 | 1.4964 | 0.2476 | n.a. | n.a. | 0.4709 |
| A1 | 0.0044 | 0.0014 | 1.4711 | 0.1276 | 0.0157 | 0.0047 | 0.2895 |
| A2 | 0.0046 | 0.0016 | 1.4832 | 0.1389 | 0.0539 | 0.0152 | 0.3070 |
| A3 | 0.0048 | 0.0017 | 1.4830 | 0.1545 | 0.0192 | 0.0055 | 0.3319 |
| A4 | 0.0050 | 0.0019 | 1.4972 | 0.1695 | 0.0536 | 0.0174 | 0.3543 |
| *Sensitivity analysis 8: susceptible contacts only isolated once* | | | | | | | |
| Baseline | 0.0045 | 0.0017 | 1.3781 | 0.1859 | n.a. | n.a. | 0.3491 |
| A1 | 0.0036 | 0.0008 | 1.3742 | 0.0801 | 0.0152 | 0.0032 | 0.1764 |
| A2 | 0.0038 | 0.0010 | 1.3958 | 0.0961 | 0.0523 | 0.0129 | 0.2080 |
| A3 | 0.0038 | 0.0010 | 1.3737 | 0.1087 | 0.0194 | 0.0046 | 0.2266 |
| A4 | 0.0040 | 0.0012 | 1.3852 | 0.1217 | 0.0544 | 0.0133 | 0.2460 |

Table S11: Simulation results for the school setting - additional sensitivity analyses - **no heterogeneity in transmission**. Baseline = no quarantine, A1 = 21 days quarantine, A2 = 18 days quarantine, A3 = quarantine from day 9 to day 21, A4 = quarantine from day 9 to day 18.

| **Scenario** | $FS_{\mathbf{all}}$ | $FS_{\mathbf{newWave}}$ | $R_{\mathbf{index}}$ | $P_{\mathbf{onward}}$ | $P_{\mathbf{infQ}}$ | $P_{\mathbf{onwardQ}}$ | $P_{\mathbf{newWave}}$ |
|---|---|---|---|---|---|---|---|
| Baseline | 0.0041 | 0.0013 | 1.3879 | 0.1329 | n.a. | n.a. | 0.2634 |
| A1 | 0.0039 | 0.0011 | 1.3606 | 0.1193 | 0.0018 | 0.0002 | 0.2330 |
| A2 | 0.0040 | 0.0012 | 1.3815 | 0.1236 | 0.0065 | 0.0007 | 0.2474 |
| A3 | 0.0039 | 0.0011 | 1.3754 | 0.1222 | 0.0028 | 0.0005 | 0.2434 |
| A4 | 0.0039 | 0.0012 | 1.3630 | 0.1210 | 0.0057 | 0.0006 | 0.2385 |
| *Sensitivity analysis 1: quarantine even after PEV* | | | | | | | |
| Baseline | 0.0041 | 0.0012 | 1.3812 | 0.1319 | n.a. | n.a. | 0.2551 |
| A1 | 0.0036 | 0.0008 | 1.3771 | 0.0761 | 0.0112 | 0.0013 | 0.1689 |
| A2 | 0.0036 | 0.0008 | 1.3633 | 0.0837 | 0.0380 | 0.0051 | 0.1804 |
| A3 | 0.0038 | 0.0010 | 1.3851 | 0.0999 | 0.0928 | 0.0125 | 0.2078 |
| A4 | 0.0041 | 0.0013 | 1.3848 | 0.1308 | 0.1493 | 0.0290 | 0.2553 |
| *Sensitivity analysis 2: 20% of children immune, no quarantine after PEV* | | | | | | | |
| Baseline | 0.0494 | 0.0279 | 10.8634 | 0.1068 | n.a. | n.a. | 0.8337 |
| A1 | 0.0501 | 0.0286 | 10.8740 | 0.0932 | 0.0038 | 0.0009 | 0.7740 |
| A2 | 0.0536 | 0.0322 | 10.8742 | 0.1000 | 0.0145 | 0.0034 | 0.7971 |
| A3 | 0.0511 | 0.0297 | 10.8234 | 0.1025 | 0.0071 | 0.0021 | 0.8084 |
| A4 | 0.0528 | 0.0313 | 10.8703 | 0.1075 | 0.0108 | 0.0029 | 0.8316 |
| *Sensitivity analysis 3: 20% of children immune, quarantine even after PEV* | | | | | | | |
| Baseline | 0.0491 | 0.0277 | 10.8230 | 0.1076 | n.a. | n.a. | 0.8336 |
| A1 | 0.0392 | 0.0173 | 10.9385 | 0.0421 | 0.0211 | 0.0068 | 0.5021 |
| A2 | 0.0493 | 0.0279 | 10.8392 | 0.0662 | 0.0837 | 0.0243 | 0.6289 |
| A3 | 0.0729 | 0.0520 | 10.8292 | 0.0962 | 0.1854 | 0.0389 | 0.7634 |
| A4 | 0.1159 | 0.0960 | 10.9020 | 0.1297 | 0.2886 | 0.0607 | 0.8888 |
| *Sensitivity analysis 4: 70% of children immune, no quarantine after PEV* | | | | | | | |
| Baseline | 0.0188 | 0.0102 | 4.2775 | 0.1619 | n.a. | n.a. | 0.6708 |
| A1 | 0.0180 | 0.0094 | 4.2762 | 0.1374 | 0.0031 | 0.0007 | 0.6139 |
| A2 | 0.0180 | 0.0094 | 4.2568 | 0.1415 | 0.0113 | 0.0021 | 0.6135 |
| A3 | 0.0185 | 0.0098 | 4.2959 | 0.1483 | 0.0049 | 0.0012 | 0.6461 |
| A4 | 0.0183 | 0.0098 | 4.2384 | 0.1486 | 0.0106 | 0.0024 | 0.6329 |
| *Sensitivity analysis 5: 70% of children immune, quarantine even after PEV* | | | | | | | |
| Baseline | 0.0190 | 0.0105 | 4.2369 | 0.1631 | n.a. | n.a. | 0.6725 |
| A1 | 0.0135 | 0.0048 | 4.2887 | 0.0653 | 0.0184 | 0.0036 | 0.3848 |
| A2 | 0.0146 | 0.0059 | 4.2789 | 0.0822 | 0.0707 | 0.0147 | 0.4365 |
| A3 | 0.0175 | 0.0089 | 4.2816 | 0.1151 | 0.1593 | 0.0317 | 0.5307 |
| A4 | 0.0243 | 0.0158 | 4.2482 | 0.1698 | 0.2601 | 0.0632 | 0.6731 |
| *Sensitivity analysis 6: 70% of children immune + 3 introductions at time 0 + quarantine after PEV* | | | | | | | |
| Baseline | 0.0285 | 0.0210 | 3.7831 | 0.0905 | n.a. | n.a. | 0.6820 |
| A1 | 0.0256 | 0.0182 | 3.7565 | 0.0296 | 0.0201 | 0.0053 | 0.2897 |
| A2 | 0.0274 | 0.0199 | 3.7615 | 0.0492 | 0.0743 | 0.0176 | 0.4126 |
| A3 | 0.0310 | 0.0237 | 3.7535 | 0.0823 | 0.1674 | 0.0355 | 0.5753 |
| A4 | 0.0388 | 0.0314 | 3.7929 | 0.1325 | 0.2703 | 0.0656 | 0.7572 |
| *Sensitivity analysis 7: 70% of children immune + one introduction every 10 days + quarantine after PEV* | | | | | | | |
| Baseline | 0.1718 | 0.1671 | 2.8215 | 0.0571 | n.a. | n.a. | 0.9931 |
| A1 | 0.1653 | 0.1610 | 2.5779 | 0.0189 | 0.0201 | 0.0034 | 0.7364 |
| A2 | 0.1670 | 0.1627 | 2.5976 | 0.0333 | 0.0728 | 0.0135 | 0.8929 |
| A3 | 0.1684 | 0.1641 | 2.5879 | 0.0491 | 0.1607 | 0.0222 | 0.9565 |
| A4 | 0.1720 | 0.1677 | 2.6210 | 0.0771 | 0.2601 | 0.0409 | 0.9949 |

Table S12: Simulation results for the school setting, including post-exposure vaccination (PEV) - **no heterogeneity in transmission**. Baseline = no quarantine, A1 = 21 days quarantine, A2 = 18 days quarantine, A3 = quarantine from day 9 to day 21, A4 = quarantine from day 9 to day 18.

| Scenario | $FS_{\textbf{all}}$ | $FS_{\textbf{newWave}}$ | $R_{\textbf{index}}$ | $P_{\textbf{onward}}$ | $P_{\textbf{infQ}}$ | $P_{\textbf{onwardQ}}$ | $P_{\textbf{newWave}}$ |
|---|---|---|---|---|---|---|---|
| Baseline | 0.2159 | 0.1492 | 3.2874 | 0.3153 | 0.0000 | 0.0000 | 0.7801 |
| A1 | 0.1021 | 0.0216 | 3.2632 | 0.0557 | 0.0261 | 0.0101 | 0.1990 |
| A2 | 0.1164 | 0.0386 | 3.2431 | 0.1001 | 0.0846 | 0.0391 | 0.3446 |
| A3 | 0.1351 | 0.0592 | 3.2777 | 0.1479 | 0.0404 | 0.0237 | 0.4334 |
| A4 | 0.1485 | 0.0750 | 3.2553 | 0.1844 | 0.0800 | 0.0485 | 0.5292 |
| *Sensitivity analysis 1:* $R_0 = 18$ | | | | | | | |
| Baseline | 0.2278 | 0.1424 | 4.0650 | 0.2730 | 0.0000 | 0.0000 | 0.8056 |
| A1 | 0.1232 | 0.0225 | 4.0710 | 0.0506 | 0.0271 | 0.0111 | 0.2097 |
| A2 | 0.1419 | 0.0430 | 4.1097 | 0.0944 | 0.0912 | 0.0411 | 0.3819 |
| A3 | 0.1610 | 0.0661 | 4.0896 | 0.1453 | 0.0431 | 0.0250 | 0.4979 |
| A4 | 0.1760 | 0.0840 | 4.0678 | 0.1813 | 0.0823 | 0.0487 | 0.6016 |
| *Sensitivity analysis 2: no immune infants, 70% of 1-3yo immune* | | | | | | | |
| Baseline | 0.3773 | 0.2910 | 5.3489 | 0.2727 | 0.0000 | 0.0000 | 0.8190 |
| A1 | 0.1781 | 0.0538 | 5.3153 | 0.0562 | 0.0274 | 0.0120 | 0.2517 |
| A2 | 0.2145 | 0.0984 | 5.3191 | 0.1043 | 0.0972 | 0.0449 | 0.4515 |
| A3 | 0.2680 | 0.1632 | 5.3462 | 0.1689 | 0.0451 | 0.0294 | 0.5625 |
| A4 | 0.2995 | 0.2023 | 5.3022 | 0.2087 | 0.0767 | 0.0536 | 0.6663 |
| *Sensitivity analysis 3: longer incubation period (median 14 days)* | | | | | | | |
| Baseline | 0.2175 | 0.1483 | 3.3888 | 0.3119 | 0.0000 | 0.0000 | 0.7843 |
| A1 | 0.1073 | 0.0260 | 3.3188 | 0.0678 | 0.0496 | 0.0196 | 0.2435 |
| A2 | 0.1320 | 0.0521 | 3.3919 | 0.1333 | 0.1396 | 0.0675 | 0.4490 |
| A3 | 0.1238 | 0.0455 | 3.3013 | 0.1154 | 0.0525 | 0.0273 | 0.3562 |
| A4 | 0.1489 | 0.0724 | 3.3610 | 0.1752 | 0.1277 | 0.0706 | 0.5385 |

Table S13: Simulation results for the daycare setting - **no heterogeneity in transmission**. Baseline = no quarantine, A1 = 21 days quarantine, A2 = 18 days quarantine, A3 = quarantine from day 9 to day 21, A4 = quarantine from day 9 to day 18

## Supplementary Figures

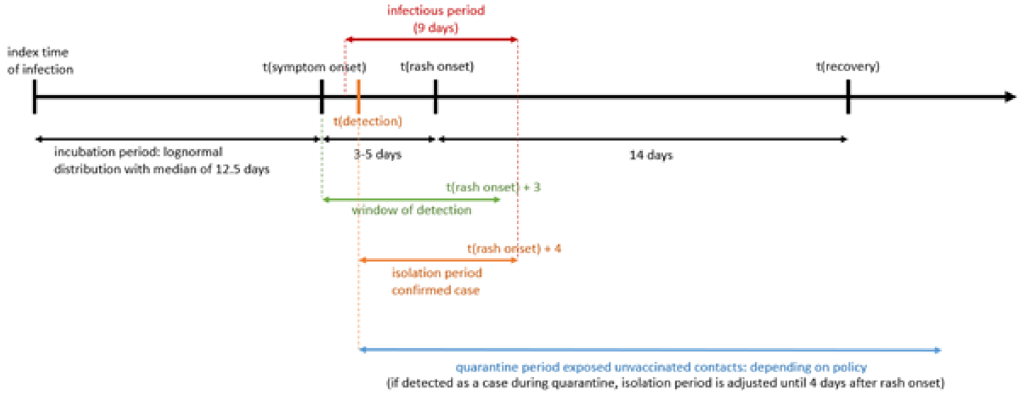


Figure S1: Natural history of measles infection and assumptions made in the simulation model.

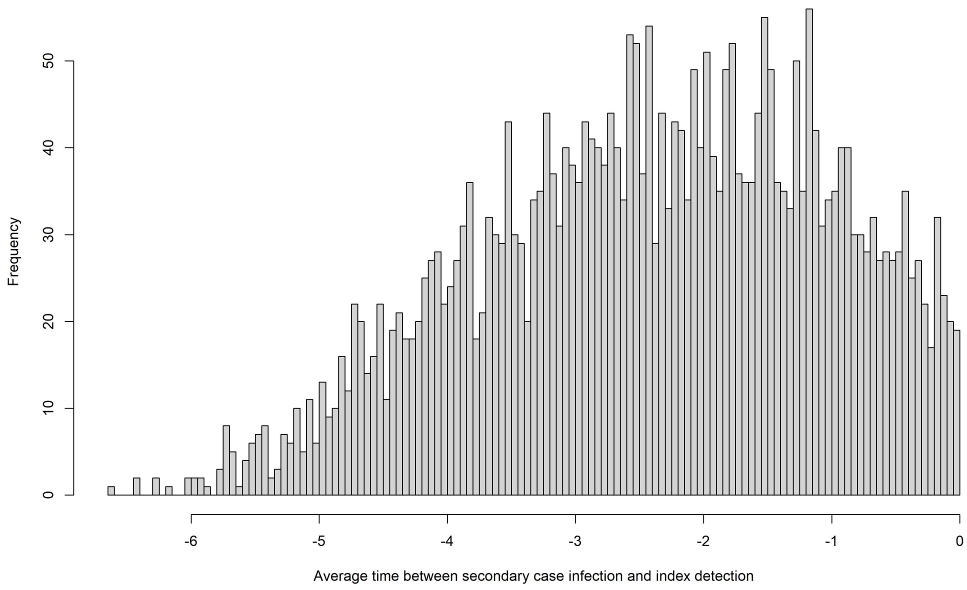


Figure S2: Average time between secondary case infection and detection of index case across 10 000 simulations in the Baseline scenario in primary school with high immunity level.

# References


1. Centers for Disease Control and Prevention. *Epidemiology and prevention of vaccine-preventable diseases: measles* [*Chapter 13, The Pink Book*] 14th ed. (CDC, 2021).
2. Lessler, J. *et al.* Incubation periods of acute respiratory viral infections: a systematic review. *The Lancet infectious diseases* **9,** 291–300 (2009).
3. Centers for Disease Control and Prevention. Measles. Annual Epidemiological Report for 2024. Surveillance Report. `https://www.ecdc.europa.eu/en/publications-data/measles-annual-epidemiological-report-2024` (2025).
4. Arciuolo, R. J., Jablonski, R. R., Zucker, J. R. & Rosen, J. B. Effectiveness of measles vaccination and immune globulin post-exposure prophylaxis in an outbreak settingâNew York City, 2013. *Clinical Infectious Diseases* **65,** 1843–1847 (2017).
5. Hoang, T. *et al.* A systematic review of social contact surveys to inform transmission models of close-contact infections. *Epidemiology (Cambridge, Mass.)* **30,** 723 (2019).
6. Guerra, F. M. *et al.* The basic reproduction number (R0) of measles: a systematic review. *The Lancet Infectious Diseases* **17,** e420–e428 (2017).
7. Blumberg, S. *et al.* Assessing measles transmission in the United States following a large outbreak in California. *PLoS currents* **7** (2015).
8. Hens, N. *et al.* Assessing the risk of measles resurgence in a highly vaccinated population: Belgium anno 2013. *Eurosurveillance* **20** (2015).
